# Visit-to-visit HbA1c and glucose variability and the risks of macrovascular and microvascular events in the general population

**DOI:** 10.1038/s41598-018-37834-7

**Published:** 2019-02-04

**Authors:** Ji-Yong Jang, Shinje Moon, Sungsoo Cho, Kyoo Ho Cho, Chang-Myung Oh

**Affiliations:** 1 Department of Internal Medicine, Chungju Medical Center, Chungju-si, South Korea; 2 Department of Endocrinology and Metabolism, Hallym University College of Medicine, Chuncheon, South Korea; 3 Department of Internal Medicine, Dankook University Hospital, Dankook University School of Medicine, Cheonan-si, South Korea; 4 Department of Neurology, Yonsei University College of Medicine, Seoul, South Korea; 5 Department of Endocrinology and Metabolism, CHA Bundang Medical Center, School of Medicine CHA University, Seongnam, South Korea

## Abstract

This study evaluate association between glycemic variability and adverse vascular events in nondiabetic middle-aged adults. From 10,020 Ansung-Ansan cohort, Korean Genome, and Epidemiology Study (KoGES) data. 6,462 nondiabetic adults aged <65 years was analyzed. The mean and coefficient of variation (CV) of all biannually recorded HbA1c, fasting blood glucose(FBG), and post 2 hr blood glucose (PBG) were calculated and divided into 3 groups based on tertile of CV at each measurement, respectively. Primary endpoint was composite of Macro (composite of Coronary artery disease, Myocardial infarction, Congestive heart failure or Stroke) and Microvascular event (Creatine Clearance <60 ml/min/1.73 m^2^). The participants (mean age: 50 years, 50% men) were followed for a median of 9.9 (9.1–10.0) years. The high HbA1c-CV tertile (odds ratio 1.30; 1.01–1.66) was independent risk factor for microvascular events. In contrast, high FBG-CV tertile (2.32; 1.30–4.12) and PBG-CV (1.85; 1.05–3.26) was for macrovascular events. In this 10-year prespective cohort study, higher HbA1c-CV tertile was associated with higher composite of macro- and microvascular events and independent risk factor in non-DM middle-aged participants. In addition, higher tertile of FBG-CV and PBG-CV were risk factors for macrovascular events.

## Introduction

Diabetes mellitus (DM) is increasing worldwide^1^, and DM-related complications, including macrovascular and microvascular complications, have created significant health and social burdens. Several recent randomized trials have evaluated the safety and efficacy of intensive glucose control for minimizing DM-related deaths and cardiovascular complications, although they have yielded controversial results^2–4^. Thus, traditional DM control based on glycated haemoglobin (HbA1c) levels may not be sufficient to predict long-term cardiovascular complications.

Recent observational studies have indicated that glycaemic variability might play an important role in the microvascular and macrovascular complications of type 1 and type 2 DM^5,6^. However, those studies were limited by their inconsistent methods for assessing variability, small sample sizes, or short follow-up durations. It is also unclear whether clinically significant glycaemic variability can be detected in non-diabetic individuals or individuals with pre-DM. Therefore, the present study evaluated long-term follow-up data from a large cohort of middle-aged Korean adults without diabetes at baseline, in order to assess whether glycaemic variability (based on variability in HbA1c and glucose levels) contributed to the development of vascular complications in this population.

## Results

### Baseline characteristics

Among the 10,030 individuals in the target cohort, 1,784 individuals were excluded because they only completed a single HbA1c test. In addition, individuals were excluded if they were >65 years old at baseline (n = 983) or fulfilled the definition of DM at baseline (n = 801) (Supplemental Fig. 1). Thus, data from 6,462 individuals were assessed to determine the coefficients of variability (CVs) for their HbA1c levels. These values were used to group the patients into the first HbA1c-CV tertile (n = 2,136), the second HbA1c-CV tertile (n = 2,199), and the third HbA1c-CV tertile (n = 2,127).

The subjects were followed for a median interval of 9.9 years (interquartile range: 9.1–10.0 years). Their clinical, biochemical, and anthropometric characteristics stratified according to CV tertile are summarized in Table 1. The mean age was 50 years and 50% of the subjects were men. The high variability groups had a greater likelihood of having a history of hypertension, although the other clinical variables were well balanced. Among the laboratory variables, the third tertile group had a higher mean HbA1c level than the other tertiles, as well as higher mean and percentage of third CV tertile group for both FBG and PBG. The trend of higher mean values for HbA1c, fasting blood glucose (FBG), and post 2-h blood glucose (PBG) according to increasing HbA1c-CV grouping was consistently observed at each visit (Supplemental Table 1). The incremental increases in HbA1c levels and newly diagnosed DM based on HbA1c levels through the follow-up were also greater in the higher HbA1c-CV groups. The third HbA1c-CV tertile group also exhibited increased insulin resistance but similar beta-cell function, relative to the other tertiles. The highest HbA1c-CV tertile also had a higher incidence of metabolic syndrome, a lower muscle mass adjusted for body mass index (BMI), and a similar fat mass.

**Table 1:** Baseline clinical and laboratory characteristics of the study population.

	HbA1c variability groups*			
	1^st^ tertile (n = 2,136)	2^nd^ tertile (n = 2,199)	3^rd^ tertile (n = 2,127)	*p*
HbA1c coefficient of variation	0.0216	0.0392	0.0659	<0.001
Age, years	50 ± 7.5	50 ± 7.5	50 ± 7.7	0.27
Male sex	1,011 (47.3)	1,056 (48.0)	1,004 (47.2)	0.84
Current smoking	463 (22.0)	463 (21.3)	447 (21.2)	0.66
Hypertension	219 (10.3)	243 (11.1)	300 (14.1)	<0.001
Dyslipidaemia	46 (2.2)	54 (2.5)	57 (2.7)	0.53
Previous MI	13 (0.6)	10 (0.5)	17 (0.8)	0.35
Previous CAD	9 (0.4)	12 (0.5)	15 (0.7)	0.46
Previous CKD	60 (2.8)	58 (2.6)	63 (3.0)	0.81
**Laboratory variables**				
Total cholesterol, mg/dL	197 ± 34	197 ± 35	197 ± 36	0.73
Triglycerides, mg/dL	142 ± 98	143 ± 99	149 ± 106	0.09
HDL-cholesterol, mg/dL	50 ± 12	50 ± 12	50 ± 12	0.94
LDL-cholesterol, mg/dL	118 ± 33	119 ± 333	117 ± 34	0.24
HbA1c, %	5.5 ± 0.3	5.5 ± 0.3	5.6 ± 0.4	<0.001
HbA1c, mmol/mol	37 ± 2	37 ± 2	38 ± 3	
High sensitivity CRP, mg/L	1.48 ± 2.74	1.50 ± 2.38	1.60 ± 3.17	0.28
Homocysteine, µmol/L	11.5 ± 3.7	11.8 ± 4.4	11.8 ± 4.4	0.053
Fasting glucose, mg/dL	91 ± 8	91 ± 8	93 ± 10	<0.001
3^rd^ tertile FBG group	596 (28.1)	680 (31.1)	845 (40.0)	<0.001
Post-prandial glucose, mg/dL	125 ± 30	126 ± 31	135 ± 39	<0.001
3^rd^ tertile PBG group	634 (29.9)	707 (32.3)	771 (36.8)	<0.001
Fasting insulin, µIU/mL	7.6 ± 4.4	7.5 ± 4.7	7.5 ± 4.5	0.77
HOMA-IR^†^	1.79 ± 0.67	1.80 ± 0.65	1.94 ± 0.90	<0.001
HOMA β-cell	108.4 ± 41.9	106.3 ± 42.1	106.9 ± 44.6	0.25
QUICKI^‡^	0.354 ± 0.018	0.353 ± 0.017	0.350 ± 0.019	<0.001
QUICKI < 0.339	412 (19.3)	416 (19.0)	575 (27.1)	<0.001
Metabolic syndrome	374 (17.5)	363 (16.5)	428 (21.0)	0.006
**Body composition variables**				
BMI, kg/m^2^	24.4 ± 3.0	24.4 ± 2.8	26.6 ± 3.1	0.02
Muscle mass, kg	43.7 ± 8.1	44.0 ± 7.9	44.1 ± 8.0	0.33
Muscle mass/BMI, m^2^	1.80 ± 0.33	1.81 ± 0.33	1.78 ± 0.33	0.01
Fat mass, kg	16.8 ± 5.3	16.7 ± 5.1	17.1 ± 5.4	0.08
Fat mass/BMI, m^2^	0.68 ± 0.16	0.68 ± 0.16	0.68 ± 0.16	0.42

Values are presented as number (%) for categorical variables and mean ± standard deviation (SD) for continuous variables.

CAD, coronary artery disease; CKD, chronic kidney disease; CRP, C-reactive protein; FBG, fasting blood glucose; HbA1c, glycated haemoglobin; HDL, high-density lipoprotein; HOMA-IR, homeostatic model assessment of insulin resistance; LDL, low-density lipoprotein; MI, myocardial infarction; OGTT, oral glucose tolerance test; PBG, post 2-h blood glucose; QUICKI, quantitative insulin sensitivity check index.

*HbA1c variability was assessed using the coefficient of variation for all HbA1c measurements throughout the study. ^†^HOMA-IR = (fasting insulin, µIU/mL) × (fasting glucose, mmol/L)/22.5; HOMA β-cell = [20 × (fasting insulin, µIU/mL)]/[(fasting glucose, mmol/L) − 3.5] (reference 15). ^‡^QUICKI = 1/[log (fasting insulin, µIU/mL) + log (fasting glucose, mg/dL)] (ref.^16^).

### Incidences of the primary and secondary outcomes according to glycaemic variability

**Figure 1:**
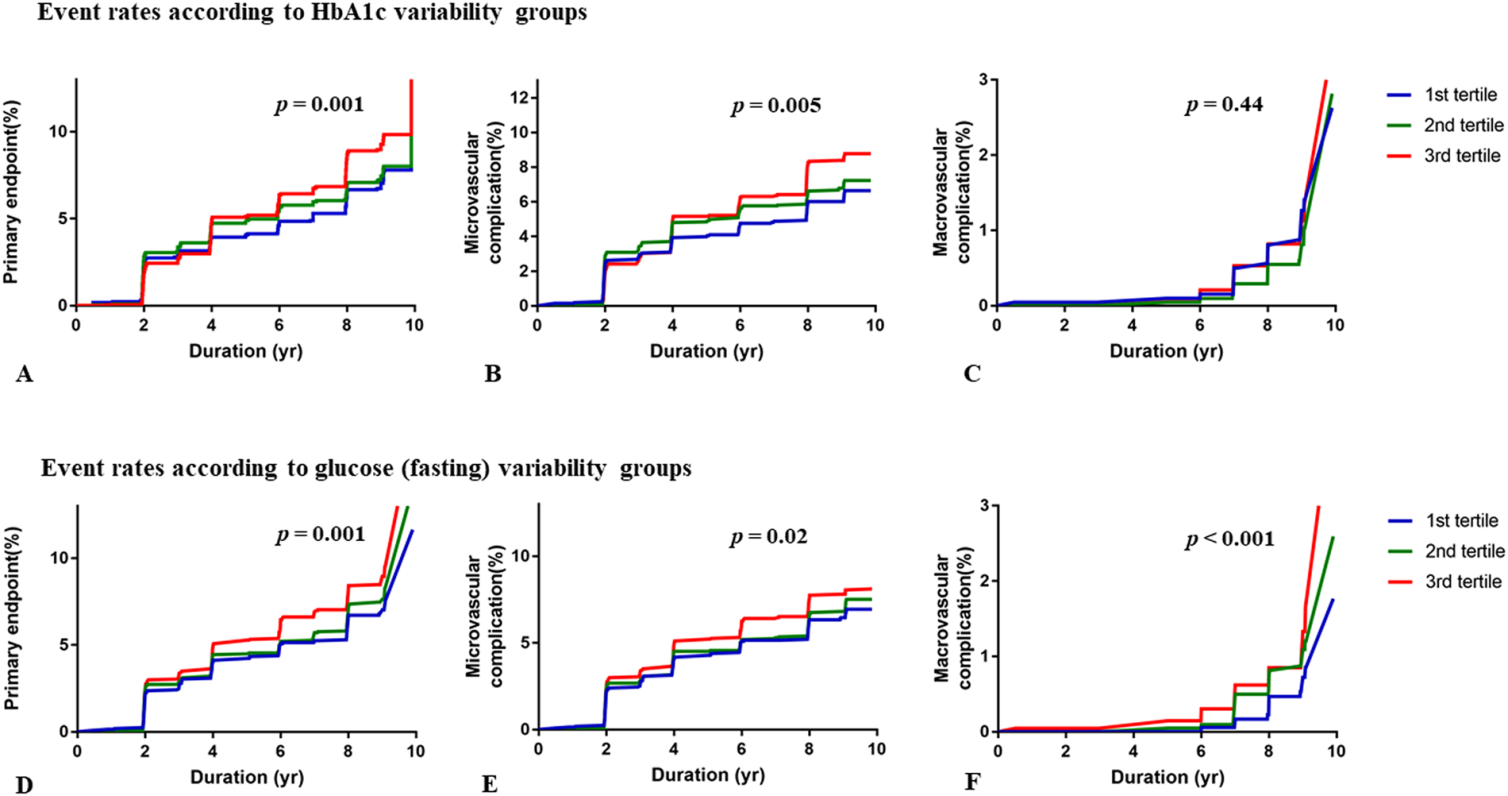
Event rates for the primary* and secondary outcomes according to the HbA1c and fasting glucose variability groups. *The primary outcome was a composite of macrovascular events (coronary artery disease, myocardial infarction, congestive heart failure, or stroke) and microvascular events (a creatinine clearance rate of <60 mL/min/1.73 m^2^). The secondary outcome were each of the macrovascular and microvascular events.

The primary outcome (a composite of macrovascular and microvascular events) was recorded in 758 cases (11.7%), including 142 macrovascular events and 649 microvascular events. The 10-year cumulative incidences of the primary outcome were 9.4% in the first HbA1c-CV tertile, 12.0% in the second HbA1c-CV tertile, and 13.8% in the third HbA1c-CV tertile (log-rank p = 0.001). A similar trend was observed for microvascular events, but not for macrovascular events (Table 2, Fig. 1). The 10-year cumulative incidences of the primary outcome were 9.2% in the first FBG-CV tertile, 11.9% in the second FBG-CV tertile, and 14.1% in the third FBG-CV tertile (p = 0.001). However, there was no significant difference in the incidence of the primary outcome according to PBG-CV tertile.

**Table 2:** Clinical outcomes.

	HbA1c variability groups*			
	1^st^ tertile (n = 2,136)	2^nd^ tertile (n = 2,199)	3^rd^ tertile (n = 2,127)	*p* ^†^
Primary outcomes^‡^	201 (9.4)	263 (12.0)	294 (13.8)	0.001
Microvascular events	167 (7.9)	231 (10.7)	251 (12.0)	0.005
Macrovascular events	39 (1.8)	49 (2.3)	54 (2.5)	0.44
CAD, MI, or CHF	21 (1.0)	33 (1.5)	34 (1.5)	0.40
Ischemic stroke	18 (0.8)	17 (0.8)	21 (1.0)	0.62
	Fasting glucose variability groups*			
	**1** ^**st**^ **tertile (n = 2,121)**	**2** ^**nd**^ **tertile (n = 2,185)**	**3** ^**rd**^ **tertile (n = 2,121)**	***p*** ^**†**^
Primary outcomes^‡^	195 (9.2)	260 (11.9)	300 (14.1)	0.001
Microvascular events	176 (7.9)	228 (10.6)	242 (11.6)	0.02
Macrovascular events	26 (1.3)	45 (2.1)	71 (3.4)	<0.001
CAD, MI, or CHF	16 (0.8)	27 (1.2)	45 (2.1)	0.002
Ischemic stroke	10 (0.5)	19 (0.9)	27 (1.3)	0.03
	Post 2-h glucose variability groups*			
	**1** ^**st**^ **tertile (n = 2,112)**	**2** ^**nd**^ **tertile (n = 2,176)**	**3** ^**rd**^ **tertile (n = 2,112)**	***p*** ^**†**^
Primary outcomes^‡^	237 (11.2)	269 (12.4)	240 (11.4)	0.82
Microvascular events	217 (10.4)	221 (10.3)	199 (9.6)	0.57
Macrovascular events	26 (1.2)	61 (2.8)	55 (2.6)	0.002
CAD, MI, or CHF	51 (0.7)	38 (1.7)	35 (1.6)	0.01
Ischemic stroke	11 (0.5)	23 (1.1)	22 (1.0)	0.14

Values are presented as number (%).

CAD, coronary artery disease; MI, myocardial infarction; CHF, congestive heart failure; HbA1c, glycated haemoglobin.

*Variability was assessed using the coefficient of variation for all measurements throughout the study. ^†^*p*-values were calculated using the log-rank test. ^‡^The primary outcome was a composite of macrovascular events (coronary artery disease, myocardial infarction, congestive heart failure, or stroke) and microvascular events (a creatinine clearance rate of <60 mL/min/1.73 m^2^).

### Effects of HbA1c and glucose variability on vascular events

Table 3 lists the estimated hazard ratios (HRs) for the development of the primary outcome based on the multivariate Cox regression analyses. These analyses revealed that the primary outcome was significantly associated with increasing age, histories of hypertension and dyslipidemia, and a higher mean HbA1c level (HR: 1.72, 95% confidence interval [CI]: 1.31–2.26; p < 0.001). Furthermore, significantly elevated risks of the primary outcome were observed in the highest HbA1c-CV tertile (HR: 1.37, 95% CI: 1.08–1.70; p = 0.008) and the highest FBG-CV tertile (HR: 1.31, 95% CI: 1.05–1.68; p = 0.02). Restricted cubic spline regression with adjustment for age and sex revealed dose-dependent relationships between HbA1c-CV, FBG-CV, and the primary outcome (Supplemental Fig. 2).

**Table 3:** Cox regression analysis of the primary outcome.

Variable	Univariate analysis		Multivariate analysis	
	HR (95% CI)	*p*	HR (95% CI)	*p*
Age, years	1.08 (1.07–10.9)	<0.001	1.07 (1.06–1.08)	<0.001
Male sex	0.63 (0.54–0.73)	<0.001	0.70 (0.59–0.84)	<0.001
Hypertension	2.17 (1.82–2.58)	<0.001	1.54 (1.24–1.92)	<0.001
Myocardial infarction	2.22 (1.19–4.14)	0.01		
Coronary artery disease	1.34 (0.56–3.24)	0.51		
Dyslipidaemia	1.44 (0.97–2.15)	0.07	1.64 (1.05–2.57)	0.03
HbA1c variability 3^rd^ tertile	1.39 (1.17–1.67)	<0.001	1.37 (1.08–1.70)	0.008
Mean HbA1c, %	2.91 (2.35–3.62)	<0.001	1.72 (1.31–2.26)	<0.001
Mean FBG	1.01 (1.00–1.02)	0.04		
Mean PBG	1.01 (1.00–1.01)	<0.001		
FBG variability 3^rd^ tertile	1.45 (0.21–1.74)	<0.001	1.31 (1.05–1.68)	0.02
PBG variability 3^rd^ tertile	1.00 (0.84–1.20)	0.96		
BMI, kg/m^2^	1.06 (1.04–1.09)	<0.001		
Muscle mass/BMI, m^2^	0.40 (0.31–0.51)	<0.001		
Fat mass/BMI, m^2^	3.31 (1.95–5.61)	<0.001		
LDL cholesterol, mg/dL	1.004 (1.002–1.006)	0.001		
New-onset diabetes	1.76 (1.35–2.28)	<0.001		

HR, hazard ratio; CI, confidence interval; HbA1c, glycated haemoglobin; FBG, fasting blood glucose; PBG, post 2-h blood glucose; BMI, body mass index; LDL, low-density lipoprotein cholesterol.

### Different effects of HbA1c and glucose variability on macro and microvascular events

Multivariate Cox regression analyses were performed for the primary outcome, macrovascular events, and microvascular events. The results revealed that the mean HbA1c level and HbA1c-CV could significantly predict the risk of developing the primary outcome and microvascular events, with increasing HRs for the primary outcome at higher HbA1c-CV tertiles (p for trend = 0.02). However, HbA1c-CV was not associated with the risk of macrovascular events (Table 4). In contrast, FBG-CV significantly predicted the risk of macrovascular events (p for trend = 0.01), with higher FBG-CV not being associated with an increased risk of microvascular events and not consistently associated with the risk of developing the primary outcome. The mean PBG level and PBG-CV were associated with increased risks of macrovascular events but not the primary outcome or microvascular events.

**Table 4:** Multivariate Cox analysis of the primary and secondary outcomes*.

	Primary outcome^†^		Microvascular		Macrovascular	
	HR (95% CI)	*p*	HR (95% CI)	*p*	HR (95% CI)	*p*
Mean HbA1c	1.72 (1.30–2.26)	<0.001	2.08 (1.46–2.97)	<0.001	1.37 (0.64–2.93)	0.42
HbA1c-CV 1^st^ tertile	Reference		Reference		Reference	
2^nd^ tertile	1.34 (1.07–1.67)	0.01	1.37 (1.08–1.74)	0.01	1.18 (0.72–1.96)	0.51
3^rd^ tertile	1.36 (1.08–1.70)	0.008	1.30 (1.01–1.66)	0.04	1.17 (0.69–1.98)	0.56
P for trend		0.02		0.08		0.63
Mean FBG	0.99 (0.97–1.00)	0.052	0.98 (0.96–0.99)	0.003	1.00 (0.97–1.03)	0.79
FBG-CV 1^st^ tertile	Reference		Reference		Reference	
2^nd^ tertile	1.17 (0.95–1.49)	0.13	1.12 (0.88–1.42)	0.35	1.69 (0.93–3.05)	0.08
3^rd^ tertile	1.31 (1.05–1.68)	0.02	1.22 (0.95–1.58)	0.12	2.32 (1.30–4.12)	0.004
P for trend		0.02		0.13		0.01
Mean PBG	1.00 (0.99–1.01)	0.31	1.00 (0.99–1.01)	0.35	1.01 (1.00–1.01)	0.005
PBG-CV 1^st^ tertile	Reference		Reference		Reference	
2^nd^ tertile	1.12 (091–1.38)	0.30	1.03 (0.82–1.29)	0.78	2.07 (1.19–3.60)	0.01
3^rd^ tertile	1.03 (0.82–1.30)	0.77	0.98 (0.77–1.25)	0.87	1.85 (1.05–3.26)	0.03
P for trend		0.85		0.77		0.09

HR, hazard ratio; CI, confidence interval; HbA1c, glycated haemoglobin; CV, coefficient of variation; FBG, fasting blood glucose; PBG, post 2-h blood glucose.

^*^The Cox regression multivariate analysis included age, sex, previous hypertension, myocardial infarction, coronary artery disease, dyslipidaemia, body mass index (BMI), low-density lipoprotein cholesterol, fat mass/BMI, muscle mass/BMI, mean HbA1c, mean FBG, mean PBG, and the tertile categories of the HbA1c, FBG, and PBG variability groupings. ^†^The primary outcome was a composite of macrovascular events (coronary artery disease, myocardial infarction, congestive heart failure, or stroke) and microvascular events (a creatinine clearance rate of <60 mL/min/1.73 m^2^).

## Discussion

The present study evaluated prospectively collected data from a 10-year cohort study and revealed that, among middle-aged participants without DM at baseline, the highest HbA1c-CV tertile was associated with an elevated risk of developing the primary outcome (a composite of macrovascular and microvascular events) or microvascular events alone. In addition, the highest FBG-CV and PBG-CV tertiles were independently associated with elevated risks of macrovascular events.

Previous studies showed that HbA1c is a valid index of long-term glycaemic control and anti-diabetes treatment efficacy, with lower HbA1c levels associated with reduced risks of diabetes-related microvascular and macrovascular complications^7,8^. However, several recent randomized studies have revealed controversial results regarding whether intensive glucose control targeting HbA1c could reduce the rate of vascular complications^2–4^. One analysis from the Diabetes Control and Complications Trial revealed a higher rate of retinopathy over time in the conventional treatment group than in the intensive treatment group, despite both groups having similar average HbA1c values^9^. Thus, it is unclear whether average glycaemic measures is the most appropriate for assessing the risks of diabetes-related complications.

Glycaemic variability is emerging as a measure of glycaemic control that may also predict diabetes-related complications. For example, a recent systemic review evaluated the associations between HbA1c variability, vascular complications, and mortality among 87,641 patients with type 1 and type 2 DM in 20 studies^10^. The meta-analysis revealed that higher HbA1c variability in type 1 DM was associated with increased risks of renal disease (risk ratio: 1.56, 95% CI: 1.08–2.25) and cardiovascular events (risk ratio: 1.98, 95% CI: 1.39–2.82) and that higher HbA1c variability in type 2 DM was associated with increased risks of renal disease (risk ratio: 1.34, 95% CI: 1.15–1.57), cardiovascular events (risk ratio: 1.27, 95% CI: 1.15–1.40), and mortality (risk ratio: 1.34, 95% CI: 1.18–1.53). Relatively a few studies have evaluated the relationship between glucose variability and cardiovascular events or mortality in DM patients. The Venoa Diabetes Study revealed that, among 54–74-year-old subjects who were followed for 10 years, fasting glycaemic variability (based on the CV value) was the strongest predictor of cardiovascular events and mortality^11^. Another recent cohort study evaluated the relationships between FBG variability, cardiovascular disease, and mortality in the general population (53,607 participants, mean age: 49.1 years, 5-year follow-up), which revealed that, after adjustment for the mean FPG value and other covariates, the highest quartile of FPG variability was associated with increased risks of cardiovascular disease (+26%) and mortality (+46%), relative to the lowest quartile^12^.

The biological effects of glycaemic variability on diabetes-related vascular complications are under-investigated. One possible explanation involves the theory of metabolic memory, which promotes a mechanism of non-enzymatic glycation of cellular transduction system and excess reactive oxygen and nitrogen that leads to disturbed signal transduction and enhanced inflammatory stress^13,14^, which subsequently leads to endothelial dysfunction^15^. Another possibility involves the effects of hypoglycaemia, as hypoglycaemia-induced activation of the sympathoadrenal system leads to cardiac stress by increasing heart rate and stroke volume^16^.

The present study is the first to use prospectively collected data to examine the long-term visit-to-visit variability in HbA1c, FBG, and PBG levels, as well as their relationships with new-onset vascular complications among subjecst without diabetes. The results revealed different trends in the relationships between HbA1c and glucose variability and the various vascular events. Although we could not determine the underlying pathophysiological mechanism, it is possible that glycaemic variation could be a significant prognostic predictor in the non-diabetic state, and that the biological effects of glucose and HbA1c variation could be different. Further research is needed to address this issue, as there is no evidence regarding whether these two factors are fundamentally different factors or different characteristics of a single phenomenon. Our results suggest that HbA1c variability is a better representation of insulin resistance and its associated inflammatory response. Glucose variability may also suggest the presence of insulin resistance, but better represents the activation of the sympathoadrenal system that is associated with hypoglycaemia.

Our study also had several limitations. First, data regarding clinical events were obtained via questionnaires that were administered by a trained interviewer, and the incidence of macrovascular events in this relatively healthy cohort was lower than among people with diabetes. However, large cohort studies routinely use standardized questionnaires, and our observed incidence of macrovascular events was similar to that in other ethnic groups without diabetes^17^. Moreover, the absence of data regarding other microvascular events, such as retinopathy, is a potential limitation, although the expected incidences of end-stage DM related microvascular events would be very low, as the subjects did not have diabetes at baseline. Second, we could not evaluate all-cause mortality or cardiovascular mortality in this cohort. Third, we could not evaluate intra-day or inter-day fluctuations in serum glucose levels, although there is currently no standardized definition of HbA1c variability and most studies have expressed variability based on the standard deviation or CV for all measurements during an investigational period^10^. Fourth, we did not include dietary and medication information, which could affect clinical outcomes, as this lay outside the aim of this study. Nevertheless, it would be interesting to evaluate whether dietary or medical intervention could affect the development of future clinical events.

Recently the development of new technologies for glucose monitoring has made it possible to identify glucose variability and improve glucose control. In this context, recent studies have yielded encouraging results from the use of glucose sensors in combination with an insulin pump^18^, which suggests that glucose variability could be an important measure for validating new DM therapies, as well as for predicting the risk of DM and its vascular complications.

In conclusion, data from a 10-year prospective cohort study revealed that high HbA1c-CV in middle-aged individuals without DM at baseline was independently associated with the primary outcome (a composite of macrovascular and microvascular events) and microvascular events alone. In addition, high FBG-CV and PBG-CV values were independently associated with an increased risk of macrovascular events.

## Methods

### Study population

The epidemiological data were collected from the Ansan (urban) and Ansung (rural) prospective community-based cohort studies. These studies are part of the Korean Health and Genome Study (KoGES), which is conducted by the Korea Centers for Disease Control and Prevention (Republic of Korea) as a government-funded epidemiological survey to investigate trends in chronic non-communicable diseases and their associated risk factors^19^. The studies included 10,030 participants who were 40–69 years at baseline (2001–2002), The age-sex distributions of the study populations were similar to those of the general populations in each area. Biennial surveys, which included administered questionnaires and clinical examinations, were continued up to the sixth follow-up phase in 2014. The present study evaluated individuals who were <65 years old and did not have DM at baseline. Subjects were excluded if they had been diagnosed with type 2 DM, were taking anti-diabetes medication(s) at baseline, or had a mean HbA1c level of >6.5% (>48 mmol/mol) during the follow-up. Subjects were also excluded if they only completed a single laboratory test. Thus, a total of 6,462 individuals were included in the present study.

### Assessment of HbA1c and glucose variability, anthropometric factors, and clinical characteristics

The biennial surveys collected the following clinical, laboratory and anthropometric data: height; weight; waist circumference; blood pressure; and biochemical results, including HbA1c, FBG, insulin, lipid profile, and biomarkers reflecting systemic inflammatory status (high sensitivity C-reactive protein and homocysteine), as previously described^20^. Blood samples were obtained after an overnight fast of at least 8 h, and HbA1c levels were measured using high-performance liquid chromatography (Variant II; BioRad Laboratories, Hercules, CA, USA).

All participants also underwent a standard 75-g oral glucose tolerance test after an overnight fast^21^. All measurements were expressed as mean ± standard deviation. The fasting insulin and glucose values were used to calculate the values for the homeostasis model of assessment–insulin resistance (HOMA-IR), homeostasis model of assessment–β-cell (HOMA–β-cell)^22^, and the quantitative insulin sensitivity check index (QUICKI). A QUICKI value of <0.339 indicates insulin resistance^23,24^.

The mean, coefficient of variation (CV) of all recorded HbA1c, fasting blood glucose (FBG), and post 2 hr blood glucose (PBG) based on Oral glucose tolerance test were calculated for each person. And the CV was employed as measures of visit to visit variability in HbA1c, fasting and post 2 hr glucose. We divided population into 3 groups according to tertile of CV each value, respectively. In this study, we present baseline characteristics based on CV tertile groups of HbA1c (HbA1c-CV).

The subjects’ BMI values were calculated as weight divided by height squared (kg/m^2^). Lean body mass and body fat mass were assessed using multifrequency bioelectrical impedance analysis (MF-BIA; Inbody 3.0, Biospace, Seoul, Korea), which provides valid and accurate that are closely associated with those measured using dual-energy x-ray absorptiometry across broad ranges of age, volume status, and BMI^25^. Mean muscle and fat mass were also adjusted for mean BMI^26^.

Metabolic syndrome was diagnosed based on the presence of at least three of the Adult Treatment Panel-III risk factors: abdominal obesity (waist circumference: >102 cm for men and >88 cm for women), elevated blood pressure (a systolic pressure of ≥130 mmHg, a diastolic pressure of ≥85 mmHg, or receiving antihypertensive treatment), impaired fasting glucose (fasting plasma glucose of ≥100 mg/dL), atherogenic dyslipidaemia (triglycerides of ≥150 mg/dL or high-density lipoprotein of 40 mg/dL for men and <50 mg/dL for women)^27^.

### Outcome definitions

The primary outcome was a composite of macrovascular events (coronary artery disease, myocardial infarction, hospitalization for congestive heart failure, and ischemic stroke) and microvascular events. Previous or new-onset macrovascular events were identified based on the biennial surveys, with all reported cases confirmed through repeated in-depth personal interviews^28^. Microvascular events were identified based on a creatine clearance rate of <60 mL/min/1.73 m^2^ during the follow-up. Creatine clearance was calculated using the Modification of Diet in Renal Disease equation at each visit, and subjects with chronic kidney disease at baseline were excluded from the survival analysis. The secondary outcomes were the macrovascular and microvascular event each.

### Statistical analysis

Continuous variables were presented as mean ± standard deviation or median and interquartile range, and were compared between groups using one-way analysis of variance or the Kruskal-Wallis test, as appropriate. Categorical variables were presented as number (percentage) and compared using the χ^2^ test or Fisher’s exact test, as appropriate.

The cumulative incidences of the primary outcome were compared using the Kaplan-Meier method with the log-rank test. The Hazard Ratio(HR) and 95% Confidence interval (CI) values for the primary outcome were estimated using univariate and multivariate Cox’s proportional hazard models. The multivariate model included age; sex; hypertension; dyslipidemia; coronary artery disease; myocardial infarction; body mass index; low-density lipoprotein-cholesterol; fat mass/BMI; muscle mass/BMI; the tertiles of HbA1c-CV, FBG-CV, and PBG-CV (categorical vairables); and the mean values for HbA1c, FBG, and PBG. Multivariate Cox’s regression analyses, which included significant variables in the univariate analyses and traditional risk factors for vascular events, were also performed to assess whether the mean and CV groups of HbA1c, FBG, and PBG were independently associated with the primary and second outcomes.

Graphical relationships were evaluated using restricted cubic spline plots according to the HbA1c-CV, FBG-CV and PBG-CV groupings. All analyses were performed using IBM SPSS software (version 24.0; IBM Corp., Armonk, NY, USA) and R software (version 3.1.0; the R Foundation for Statistical Computing, Vienna, Austria). Differences were considered statistically significant at p-values of < 0.05.

### Ethical considerations

The institutional review board of Bundang CHA Hospital (South Korea) approved the study protocol (CHAMC 2016-08-017). All participants volunteered for the Ansan and Ansung studies, and provided written informed consent prior to enrolment. All participants’ records were anonymized before being accessed by the authors, and all research procedures were performed in accordance with relevant guidelines and regulations.

## Supplementary information

Supplementary figure and tables
