# Supplementary material for: Visit-to-visit HbA1c and glucose variability and the risks of macrovascular and microvascular events in the general population
Source: Sci Rep. 2019 Feb 4;9:1374. doi: 10.1038/s41598-018-37834-7 (PMC6362217; doi:10.1038/s41598-018-37834-7)
Supplement: Supplementary file 1 — Supplementary figure and tables [file 41598_2018_37834_MOESM1_ESM.docx]

**Visit-to-visit HbA1c and glucose variability and the risks of macrovascular and microvascular events in the general population**

Ji-Yong Jang, Shinje Moon, Sungsoo Cho, Kyoo Ho Cho, Chang-Myung Oh


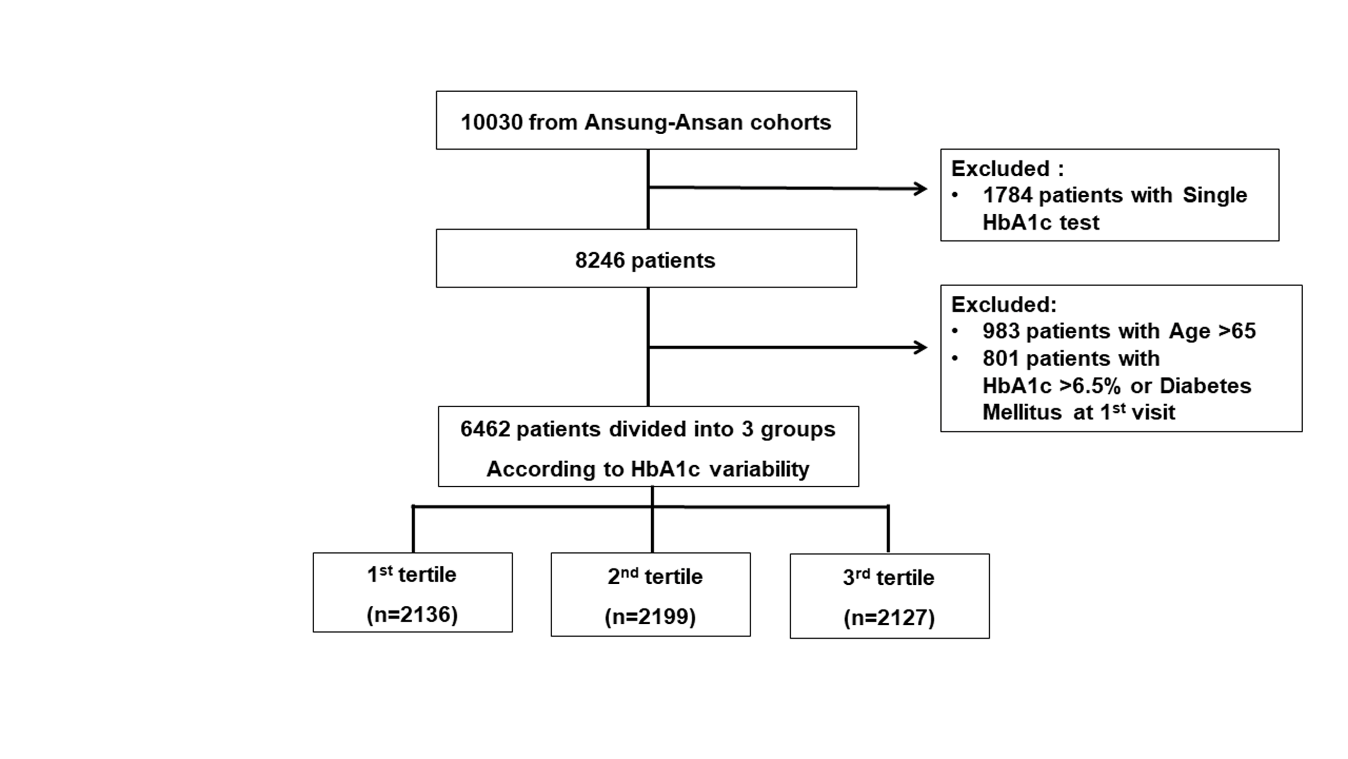
**Supplement Figure 1. Study profile.**

HbA1c variability assessed using the coefficient of variation (CV) of HbA1c measurements obtained throughout the study period. HbA1c, glycated hemoglobin

**Supplement Figure 2. The relationship between the percentile of each glucose measures and the odds ratio for primary endpoints* adjusted for age and sex. A, HbA1c-CV; B, FBG-CV; C, PBG-CV**

**
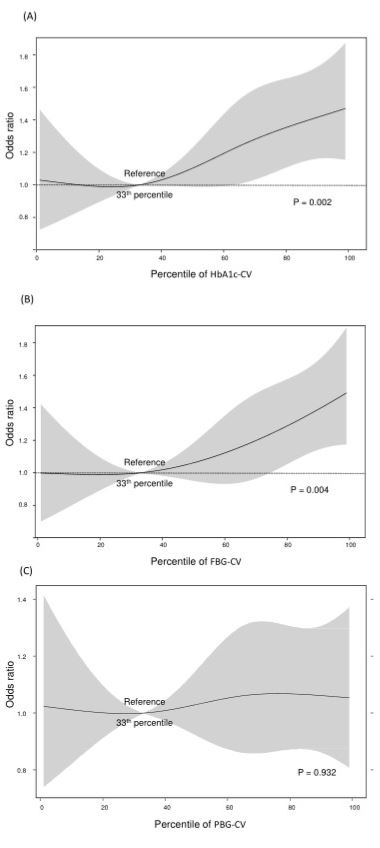
**

CV: Coefficient of Variation. *Primary endpoint was composite of Macro (composite of CAD, MI, CHF or ischemic stroke )- and Microvascular event (defined as Creatine Clearance < 60ml/min/ 1.73m^2^)

**Supplement Table 1. Serial change of Coefficient of variation(CV), HbA1c, Fasting blood glucose, post 2hour blood glucose, muscle mass and fat mass through follow-up duration.**

| **HbA1c variability groups** | | | | |  |
| --- | --- | --- | --- | --- | --- |
| **HbA1c, %** | **1^st^ tertile** | **2^nd^ tertile** | **3^rd^ tertile** | **p** | |
| New onset DM, n(%) | 6 (0.3) | 26 (1.2) | 254 (11.9) | <0.001 | |
| Visit 1 (mean±SD, (CV)) | 5.5±0.3 (0.056) | 5.5±0.3 (0.059) | 5.6±0.4 (0.076) | <0.001 | |
| Visit 2 | 5.7±0.3 (0.060) | 5.7±0.3 (0.058) | 5.8±0.5 (0.096) | 0.35 | |
| Visit 3 | 5.4±0.3 (0.056) | 5.4±0.3 (0.062) | 5.4±0.4 (0.082) | <0.001 | |
| Visit 4 | 5.5±0.3 (0.056) | 5.5±0.4 (0.066) | 5.5±0.5 (0.088) | 0.23 | |
| Visit 5 | 5.5±0.3 (0.075) | 5.6±0.4 (0.065) | 5.7±0.5 (0.096) | <0.001 | |
| Visit 6 | 5.5±0.3 (0.074) | 5.5±0.4 (0.065) | 5.7±0.5 (0.093) | <0.001 | |
| *Difference between  Visit 1 and Visit 6 | 0.00±0.17 | 0.01±0.29 | 0.11±0.55 | <0.001 | |
|  |  |  |  |  | |
| **FBG, mg/dL** | **1^st^ tertile** | **2^nd^ tertile** | **3^rd^ tertile** | **p** | |
| Visit 1 | 87±9 | 87±9 | 88±10 | <0.001 | |
| Visit 2 | 90±10 | 90±10 | 91±10 | 0.001 | |
| Visit 3 | 90±9 | 90±9 | 91±10 | <0.001 | |
| Visit 4 | 91±9 | 92±10 | 94±13 | <0.001 | |
| Visit 5 | 94±9 | 95±10 | 98±14 | <0.001 | |
| Visit 6 | 93±9 | 94±9 | 97±14 | <0.001 | |
| *Difference between  Visit 1 and Visit 6 | 6±10 | 7±10 | 8±12 | <0.001 | |
|  |  |  |  |  | |
| **PBG, mg/dL** | **1^st^ tertile** | **2^nd^ tertile** | **3^rd^ tertile** | **p** | |
| Visit 1 | 120±32 | 121±34 | 128±40 | <0.001 | |
| Visit 2 | 119±34 | 120±35 | 124±38 | <0.001 | |
| Visit 3 | 123±37 | 124±37 | 131±44 | <0.001 | |
| Visit 4 | 128±38 | 127±38 | 137±47 | <0.001 | |
| Visit 5 | 126±36 | 127±38 | 137±49 | <0.001 | |
| Visit 6 | 126±36 | 127±38 | 136±49 | <0.001 | |
| *Difference between  Visit 1 and Visit 6 | 7±36 | 8±37 | 12±45 | <0.001 | |
| **Muscle mass /BMI, m^2^** | **1^st^ tertile** | **2^nd^ tertile** | **3^rd^ tertile** | **p** | |
| Visit 1 | 1.80±0.33 | 1.81±0.32 | 1.80±0.32 | 0.53 | |
| Visit 2 | 1.82±0.33 | 1.83±0.33 | 1.81±0.33 | 0.18 | |
| Visit 3 | 1.82±0.34 | 1.83±0.33 | 1.81±0.33 | 0.11 | |
| Visit 4 | 1.82±0.34 | 1.82±0.33 | 1.79±0.33 | 0.02 | |
| Visit 5 | 1.80±0.33 | 1.81±0.33 | 1.78±0.33 | 0.01 | |
| Visit 6 | 1.78±0.33 | 1.80±0.34 | 1.76±0.33 | 0.006 | |
| *Difference between  Visit 1 and Visit 6 | -0.009±0.091 | -0.015±0.091 | -0.018±0.097 | 0.07 | |
|  |  |  |  |  | |
| **Fat mass/BMI, m^2^** | **1^st^ tertile** | **2^nd^ tertile** | **3^rd^ tertile** | **p** | |
| Visit 1 | 0.67±0.15 | 0.67±0.15 | 0.68±0.15 | 0.32 | |
| Visit 2 | 0.66±0.15 | 0.66±0.15 | 0.66±0.15 | 0.18 | |
| Visit 3 | 0.66±0.15 | 0.66±0.15 | 0.67±0.15 | 0.08 | |
| Visit 4 | 0.66±0.15 | 0.66±0.15 | 0.68±0.15 | 0.001 | |
| Visit 5 | 0.66±0.15 | 0.66±0.15 | 0.68±0.15 | <0.001 | |
| Visit 6 | 0.67±0.15 | 0.67±0.15 | 0.69±0.15 | <0.001 | |
| *Difference between  Visit 1 and Visit 6 | 0.003±0.087 | 0.001±0.089 | 0.003±0.094 | 0.35 | |

Values are presented as mean ± standard deviation (SD). Abbreviation: BMI, body mass index; FBG, fasting blood glucose; PBG, post 2hour blood glucose. * Difference between Visit 1 and Visit 6 was calculated by subtracted the value of Visit 1 from the value of Visit 6.

**Supplement Table2. Multivariate Cox analysis for Primary and Secondary endpoints***

|  | **Primary endpoint**^†^ | | **Microvascular** | | **Macrovascular** | |
| --- | --- | --- | --- | --- | --- | --- |
|  | HR(95% CI) | *p* | HR(95% CI) | *p* | HR(95% CI) | *p* |
| Mean HbA1c | 1.62 (1.23-2.13) | 0.001 | 2.04 (1.44-2.91) | <0.001 | 1.33(0.62-2.85) | 0.46 |
| HbA1c-CV 1^st^ tertile | Reference |  | Reference |  | Reference |  |
| 2^nd^ tertile | 1.34 (1.07-1.67) | 0.01 | 1.38(1.08-1.75) | 0.01 | 1.19(0.72-1.98) | 0.49 |
| 3^rd^ tertile | 1.36 (1.08-1.70) | 0.01 | 1.31 (1.02-1.68) | 0.04 | 1.18 (0.70-2.00) | 0.53 |
| P for trend |  | 0.01 |  | 0.03 |  | 0.76 |
| Mean FBG | 0.99 (0.97-1.00) | 0.04 | 0.98 (0.97-0.99) | 0.005 | 1.00(0.97-1.02) | 0.72 |
| FBG-CV 1^st^ tertile | Reference |  | Reference |  | Reference |  |
| 2^nd^ tertile | 1.18 (0.94-1.48) | 0.15 | 1.11 (0.88-1.41) | 0.37 | 1.69 (0.93-3.05) | 0.08 |
| 3^rd^ tertile | 1.32 (1.04-1.67) | 0.02 | 1.21 (0.94-1.56) | 0.14 | 2.32 (1.30-4.12) | 0.004 |
| P for trend |  | 0.07 |  | 0.33 |  | 0.01 |
| Mean PBG | 1.00 (0.99-1.01) | 0.32 | 1.00 (0.99-1.01) | 0.36 | 1.01(1.00-1.01) | 0.005 |
| PBG-CV 1^st^ tertile | Reference |  | Reference |  | Reference |  |
| 2^nd^ tertile | 1.10 (0.89-1.36) | 0.36 | 1.02 (0.81-1.28) | 0.88 | 2.07 (1.19-3.60) | 0.01 |
| 3^rd^ tertile | 1.02 (0.82-1.28) | 0.84 | 0.97 (0.76-1.24) | 0.82 | 1.85 (1.05-3.26) | 0.03 |
| P for trend |  | 0.63 |  | 0.93 |  | 0.03 |

HR, hazard ratio; CI, confidence interval; HbA1c, glycated haemoglobin; CV, coefficient of variation; FBG, fasting blood glucose; PBG, post 2-h blood glucose.

^*^The Cox regression multivariate analysis included age, sex, previous hypertension, myocardial infarction, coronary artery disease, dyslipidaemia, body mass index (BMI), low-density lipoprotein cholesterol, fat mass/BMI, muscle mass/BMI, mean HbA1c, mean FBG, mean PBG, and the tertile categories of the HbA1c, FBG, and PBG variability groupings and metabolic syndrome. ^†^ The primary outcome was a composite of macrovascular events (coronary artery disease, myocardial infarction, congestive heart failure, or stroke) and microvascular events (a creatinine clearance rate of <60 mL/min/1.73 m^2^).
